# Plasmapheresis Treatment of Hypertriglyceridemia-Induced Acute Pancreatitis: A Case Report

**DOI:** 10.7759/cureus.8360

**Published:** 2020-05-30

**Authors:** Foma Munoh Kenne, Emanuela Cimpeanu, Rana Al-Zakhari, George N Freg, Jay Nfonoyim

**Affiliations:** 1 Internal Medicine, Richmond University Medical Center, Staten Island, USA; 2 Pulmonary Critical, Richmond University Medical Center, Staten Island, USA

**Keywords:** plasmapheresis, hypertriglyceridemia, acute pancreatitis, mortality, ranson’s score

## Abstract

Hypertriglyceridemia is the third most common etiology for acute pancreatitis (AP), after alcohol and gallstones. Clinical evidence is relatively weak in its support of plasmapheresis for the treatment of hypertriglyceridemia-induced acute pancreatitis (HTG-AP). We report a case of severe HTG-AP in a young man who was successfully treated with plasmapheresis. The patient achieved full resolution of symptoms within 48 hours from presentation and was discharged two days later. To our knowledge, no other report in literatures shows such dramatic response to plasmapheresis.

## Introduction

The incidence of acute pancreatitis (AP) is estimated at 14% in patients with hypertriglyceridemia [1]. Hypertriglyceridemia-induced acute pancreatitis (HTG-AP) is thought to manifest with increased severity compared to pancreatitis secondary to other causes, though mortality rates have not been found to differ [2]. There is no clear evidence as to which hypertriglyceridemic patients will develop AP and as to whether they are at increased risk for chronic disease [2]. For the most part, the management of HTG-AP consists of supportive measures such as fluid resuscitation, pain control and, if indicated, antibiotics [3]. In HTG-AP, plasmapheresis has proven beneficial in reducing the level of triglycerides (TG), although there is no clearly established evidence for its benefits in decreasing mortality [1]. Herein we report a case of severe HTG-AP successfully treated with plasmapheresis.

## Case presentation

A 38-year-old male presented with a one day history of epigastric pain radiating to the back, severe nausea, and multiple episodes of emesis. His past medical history included anxiety disorder, hypertension, and chronic leg pain secondary to tethered cord syndrome. He had no history of alcohol consumption, smoking or drug abuse and had no hyperlipidemia or type II diabetes mellitus. The patient had a family history of pancreatic cancer in mother and myocardial infarction in father. He denied a family history of hypertriglyceridemia.

The patient was pale, hyperventilating, and in severe distress. Physical examination demonstrated epigastric tenderness with sluggish bowel sounds. The remainder of the exam was unremarkable for any abdominal masses, organomegaly, tendon xanthomas, xanthelasmas eruptive xanthomas, or lipemia retinalis. The patient was fully awake and alert. Vital signs were: blood pressure 148/98 mmHg, heart rate 114 bp, respiratory rate 24 bpm, oxygen saturation 95% on right atrial (RA), and temperature 101.4°F. Laboratory workup revealed white blood count 24.8 k/uL, hemoglobin 14.8 g/dL, sodium 124 mmol/L, potassium 2.4 mmol/L, chloride 82 mmol/L, serum calcium adjusted for albumin < 5.0 mg/dL, blood urea nitrogen 21 mg/dL, creatinine 3 mg/dL, lactic acid 9.0 mmol/L, TG 3484 mg/dL, lipase 3306 U/L, and unmeasurably elevated aspartate aminotransferase (AST) and alanine aminotransferase (ALT).

**Figure 1:**
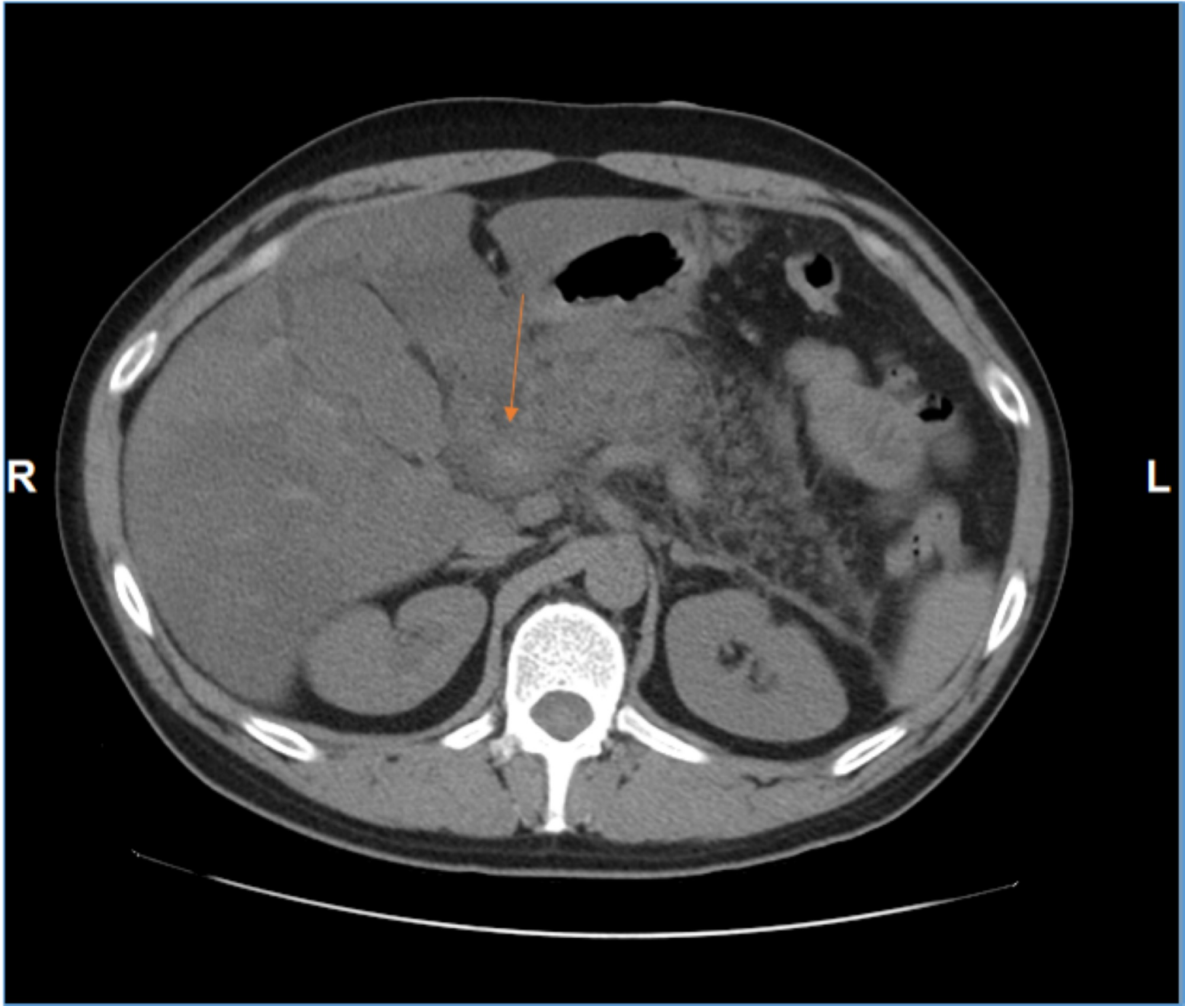
CT scan abdomen. CT scan abdomen shows that the pancreatic head is enlarged (the arrow), ill-defined and surrounded by fluid and fat stranding, consistent with acute pancreatitis. Inflammatory fluid and fat stranding throughout most of the upper abdomen. No walled fluid collections to indicate pancreatic pseudocyst or abscess.

Further evaluation with ultrasonography of the abdomen showed a diffusely enlarged pancreas consistent with acute pancreatitis, slightly distended gallbladder, hepatomegaly with diffuse hepatic steatosis and small ascites. The patient was referred for abdominal CT which revealed pancreatitis with extensive inflammatory fat stranding, as well as hepatomegaly with marked geometric fatty infiltration (Figure 1).

No evidence of necrosis, pseudocyst, or abscess formation was found. A diagnosis of HTG-AP was made. Ranson’s score on admission was 4, which indicated that the patient had severe pancreatitis.

Lactated ringer and empiric antibiotic coverage were started, as was pain control. In light of the severity of the patient’s condition, clinical decision was made to initiate plasmapheresis. After one cycle of plasmapheresis (16 hours after initial presentation), repeat TG level was 589. TG further decreased to 481 during the subsequent 12 hours. Both renal function and serum calcium normalized within one day (creatinine 0.6 mg/dL and serum calcium adjusted for albumin 8.8 mg/dL). Electrolytes improved (sodium 139 mmol/L, potassium 3.0 mmol/L) and so did liver function tests (AST 176 U/L and ALT 91 U/L). Within hours of plasmapheresis, the patient demonstrated tremendous clinical improvement, which continued throughout his hospital stay. Abdominal pain significantly lessened, and he was able to tolerate oral intake within 24 hours. He was discharged in stable condition, less than 96 hours after initial admission, on gemfibrozil 600 twice daily and statin therapy. By the time of discharge, TG level had decreased to 208. The patient remained asymptomatic and close outpatient follow-up was arranged.

## Discussion

Hypertriglyceridemia is the third most common cause of AP, after gallstones and alcohol, and the causative factor in about 9% of patients [1, 3]. It is usually seen in patients whose TG levels exceed 1000 mg/dL [3]. Studies estimate that one in five patients with severe hypertriglyceridemia will experience at least one episode of acute pancreatitis [4]. Due to hypertriglyceridemia, AP appears to be more severe than when secondary to other causes [1]. Hypertriglyceridemic patients with AP tend to be younger and the majority require ICU care [4].

The pathogenesis by which high TG level causes pancreatitis is not fully understood. It is postulated that the pancreas secretes a high concentration of lipase, which hydrolyzes TG to glycerol and free fatty acids [5]. These free fatty acids are nontoxic to the pancreas, if bound to albumin [5]. However, when TG levels are very high, albumin becomes saturated, which can lead to the accumulation of free fatty acids in the pancreas [5]. This can lead to a cytotoxic reaction as well as vascular endothelial damage, which can result in pancreatic inflammation and ischemic injury [5]. While most patients with hypertriglyceridemia have some form of primary or genetic defect in lipid metabolism, for others it is secondary to uncontrolled diabetes mellitus, obesity, hypothyroidism, end-stage renal disease, nephrotic syndrome, HIV, and medications such as isotretinoin, tamoxifen, and estrogen [6].

The mainstay therapy for HTG-AP is targeted towards decreasing lipid levels via pharmacological agents [7]. Although not a traditional approach, heparin and insulin have been successfully used towards decreasing triglycerides in HTG-AP, by mechanism of enhancing activity of lipoprotein lipase [5]. Plasmapheresis has been proposed as an alternative, nonpharmacological intervention, which can achieve immediate decrease in serum triglyceride levels and minimize risk for severe complications such as pancreatic necrosis as well as the need for ICU level of care [7]. While conservative treatment takes days or even weeks to lower serum TG levels, plasmapheresis has been proven to reduce excess serum levels in about two hours. Specifically, a single session of plasmapheresis can reduce TG by an average 70% [7]. However, evidence for its use remains inconclusive.

In a systematic review of 1340 patients with HTG-AP, while plasmapheresis has proven beneficial in reducing triglyceride levels, it had no clear benefit in decreasing mortality [1]. In patients who presented with HTG-AP and for whom double filtration plasmapheresis was implemented, dramatic reduction in triglyceride level (overall 84.7%) was achieved after either one or two sessions [3]. However, all patients had extensive hospital stays, ranging from 25 to 52 days [3]. A similar response was achieved by our patient (83% reduction after one cycle), but, contrary to the above, he experienced significant improvement in symptoms and was discharged less than 96 hours from the time of presentation.

In a study by Chen et al. on patients with severe hyperlipidemic pancreatitis (Ranson’s score ≥ 3), there was no significant difference in mortality and systemic complications in patients who received plasmapheresis versus those who did not receive this intervention [8]. Of note, for those who received plasmapheresis, there was an average delay of three days from the time of symptom onset till the start of the intervention [8]. Syed et al. showed that, while plasmapheresis resulted in significant triglyceride reduction (>70%), it had no definite benefit in decreasing the length of hospitalization or preventing complications [9]. It has been postulated that the earlier the plasma exchange is being implemented, the more favorable the results [8].

Our patient’s Ranson’s score was 5, thus indicating a case of severe pancreatitis which translates into an approximate 40% mortality [10]. Immediate utilization of plasmapheresis could explain our patient’s excellent outcome in such a short amount of time. To our knowledge, no other documented report in literature shows such immediate clinical improvement to plasmapheresis.

## Conclusions

Plasmapheresis appears to be a viable option for reducing TG level in HTG-AP. In our patient’s case, it proved highly efficacious, with impressive results being seen less than 24 hours after presentation. Given that its use has been mostly reported on small numbers of patients, further research needs to be conducted on its clinical application. 
